# Ginsenoside Rg3 promotes chemosensitivity in lung adenocarcinoma organoids via apoptotic pathways

**DOI:** 10.3389/fphar.2026.1791170

**Published:** 2026-04-01

**Authors:** Min Liu, Yanxia Li, Bing Han, Ni Zeng, Xiuhua Wei, Xiaoli Xing, Zhongmin Jiang, Xiaozhi Liu, Chunyan Zhang

**Affiliations:** 1 Department of Pathology, Tianjin Fifth Central Hospital, Tianjin, China; 2 Emergency Medicine Research Institute, Tianjin Fifth Central Hospital, Tianjin, China; 3 Tianjin Key Laboratory of Epigenetics for Organ Development of Premature Infants, Tianjin Fifth Central Hospital, Tianjin, China; 4 Department of General Surgery, Tianjin Fifth Central Hospital, Tianjin, China; 5 Department of Respiratory Medicine, Tianjin Fifth Central Hospital, Tianjin, China; 6 Department of Thoracic Surgery, Tianjin Fifth Central Hospital, Tianjin, China; 7 School of Medicine, Tianjin University, Tianjin, China

**Keywords:** chemosensitivity, Combination therapy, drug screening, lung adenocarcinoma organoids, Rg3

## Abstract

**Introduction:**

Platinum-based chemotherapy remains a cornerstone for advanced non-small cell lung cancer (NSCLC), but its efficacy is often compromised by chemoresistance, necessitating strategies to restore drug sensitivity. Ginsenoside Rg3, an active component of Panax ginseng, exhibits anti-tumor and potential chemosensitizing properties, though its mechanisms in clinically relevant models are not fully understood.

**Methods:**

We successfully established and characterized three lung adenocarcinoma patient-derived organoid (PDO) lines that faithfully recapitulated the histopathological and molecular features of the parental tumors. The chemosensitizing effect of Rg3 on cisplatin was evaluated by assessing organoid viability, half-maximal inhibitory concentration (IC50), intracellular reactive oxygen species (ROS) levels, and apoptosis via TUNEL assay.

**Results:**

Pharmacodynamic evaluation revealed that the combination of Rg3 and cisplatin exerted superior inhibitory effects on organoid viability compared to either agent alone, with a pronounced reduction in IC50. Furthermore, the combination treatment significantly increased intracellular ROS levels and induced apoptosis, as evidenced by TUNEL assay.

**Discussion:**

This study provides preclinical evidence for Rg3 as a promising chemosensitizer in lung adenocarcinoma and highlights the value of PDOs as a robust platform for personalized drug response profiling. These findings support further exploration of Rg3 as an adjunct to platinum-based chemotherapy in overcoming chemoresistance.

## 1 Background

Lung cancer is one of the malignant tumors with the highest incidence and mortality worldwide, among which non-small cell lung cancer (NSCLC) accounts for approximately 85% of all lung cancer cases (Relli et al., 2019). Although targeted and immune-based therapies have achieved remarkable advances in recent years, platinum-based chemotherapy remains a cornerstone for advanced NSCLC, particularly in patients lacking actionable driver mutations or those ineligible for immunotherapy (Megyesfalvi et al., 2023). Nevertheless, the emergence of chemotherapy resistance substantially undermines its clinical efficacy, often resulting in treatment failure and disease progression. Multiple complex mechanisms might lead to chemoresistance, such as increased drug efflux, enhanced DNA repair capacity, apoptotic pathway dysregulation, and tumor microenvironment remodeling (Zhou et al., 2019). Among these, abnormal apoptotic signaling represents a central mechanism underlying resistance. Studies have demonstrated that chemotherapeutic agents exert cytotoxic effects primarily through induction of tumor cell apoptosis, and dysregulation of key apoptotic regulators is involved (Sun et al., 2017). Consequently, pharmacologic restoration of apoptotic sensitivity has become a pivotal strategy to overcome chemotherapy resistance.

In recent years, bioactive ingredients derived from traditional Chinese medicine (TCM) have attracted increasing attention as adjuvant agents. Ginsenoside Rg3, an active component of Panax ginseng, exhibits significant anti-tumor activity through multiple pathways, including cell cycle arrest, anti-angiogenesis, immune modulation, and crucially, the induction of apoptosis (Tanko et al., 2025; Liu et al., 2024). Notably, in lung cancer cells, Rg3 has been shown to promote apoptosis and mitophagy via reactive oxygen species (ROS) production (Hwang et al., 2022). Importantly, emerging evidence suggests that Rg3 can also function as a chemosensitizer. For instance, it has been demonstrated to induce apoptosis and inhibit cell proliferation by down-regulating TIGAR in gastric precancerous lesions (Lv et al., 2022). In lung cancer, Rg3 sensitizes hypoxic cells to cisplatin by inhibiting NF-κB-mediated epithelial-mesenchymal transition (EMT) (Wang et al., 2018). Supporting its translational potential, a Chinese Herbal Formulation containing Rg3 combined with cisplatin inhibited tumor growth in patient-derived xenograft (PDX) models (Jin et al., 2017).

However, notable limitations persist. Current evidence heavily relies on conventional two-dimensional (2D) cell cultures, which, as demonstrated in comparative studies, fail to recapitulate tumor heterogeneity and often lose patient-specific drug response profiles during propagation, thereby limiting their predictive value (Kim et al., 2019). Moreover, the precise molecular mechanisms by which Rg3 sensitizes lung cancer cells to chemotherapy require systematic investigation.

In this context, PDOs offer a superior model, faithfully preserving the histoarchitecture, genomic landscape, and drug-response profiles of original tumors (Sen et al., 2022). Therefore, this study utilized the PDOs model to systematically investigate the role of Rg3 on the cisplatin sensitivity in lung adenocarcinoma.

## 2 Methods

### 2.1 Sample acquisition

Tumor tissues were obtained from lung adenocarcinoma patients undergoing surgical resection at Tianjin Fifth Central Hospital, and pleural effusion samples were collected from advanced lung adenocarcinoma patients with malignant pleural effusion. All specimens were acquired with informed consent from the patients. Tissue collection protocols and research procedures were approved by the Medical Ethics Committee of Tianjin Fifth Central Hospital (WZX-EC-KY2024025). Tumor tissue and paracancerous tissue of approximately 0.5 cm in size were dissected. A pleural effusion specimen of 100 mL was collected in a sterile bag. All samples were placed in primary tissue storage solution (bioGenous, K601005) and transported within 30 min at 4 °C to a biological safety cabinet.

### 2.2 Sample processing and organoid culture

Samples were washed three times with pre-cooled antibiotic-containing balanced salt solution, minced into approximately 1 mm^3^ pieces using ophthalmological scissors, and digested with tissue dissociation solution (Accurate International, B504) on a shaker at 37 °C for 15 min. Digestion was terminated upon observation of cell clusters, followed by filtration through a 100 μm cell strainer. The filtrate was centrifuged at 150 g for 3 min, washed twice with washing buffer, and centrifuged again. Pleural effusion-derived cells were processed through red blood cell lysis (BioGenous, E238010), centrifugation, and filtration to obtain cell suspensions. Cell viability was confirmed using trypan blue stain solution (Solarbio, C0040), only samples with ≥90% viability were used. Cell pellets were mixed 1:3 with Matrigel (Corning, 356231) for 3D embedding, and 20 μL aliquots of the cell-Matrigel mixture were seeded into a 12-well plate. The plate was then placed in an incubator at 37 °C for 15 min to allow gel polymerization, after which 1,000 μL of lung adenocarcinoma organoid medium (bioGenous, K2138-LA) was gently added to each well. Cultures were maintained in a humidified incubator at 37 °C with 5% CO_2_. The medium was replaced every 2 days, and growth status was monitored under a microscope at regular intervals. Feret diameter and counts of organoids were measured using ImageJ (Version 1.8.0).

### 2.3 Organoid passaging and cryopreservation

Passage was performed when ≥80% of the spheroids reached approximately 100 µm in diameter. The original culture medium was removed, and the cultures were gently washed with pre-cooled washing buffer. Matrigel was disrupted by pipette tip trituration, followed by incubation with organoid recovery solution (Corning, 354253) on ice for 15 min. Released organoid spheroids were pelleted at 150 g for 3 min, resuspended in dissociation reagent (bioGenous, E238001) and incubated at 37 °C. After complete dissociation, the organoids were re-embedded in 75% Matrigel and cultured as described. For banking, intact or partially dissociated organoids were recovered and centrifuged, resuspended in cryopreservation medium (bioGenous, E238023), and transferred into cryovials. They were then frozen using a controlled-rate cooling method and finally stored in liquid nitrogen.

### 2.4 Organoid hematoxylin-eosin (HE) staining

Lung adenocarcinoma organoids at passage 3 were collected using cell recovery solution, pre-stained with eosin, and embedded in 3% agarose gel. Both the organoid-gel complex and parental tissues were fixed in 4% paraformaldehyde for one hour. Following fixation, the samples were sequentially dehydrated through a graded ethanol series, cleared in xylene, infiltrated with paraffin, and embedded into paraffin blocks. Sections (4 µm thick) were cut and stained with hematoxylin-eosin. Histological morphology was observed under a light microscope (Olympus, BX53).

### 2.5 Immunohistochemical (IHC) staining

After deparaffinization and rehydration, sections (4 µm thick) were subjected to antigen retrieval using citrate buffer (pH 6.0) under high pressure for 2 min. Endogenous peroxidase activity was blocked, sections were followed by incubation with 5% goat serum at room temperature for 1 h. Primary antibodies, including Anti-Ki67 (Cat. No.ZM-0166); Anti-CK7 (Cat. No.ZM-0071); Anti-TTF1 (Cat. No. ZM-0270); Anti-Napsin A (Cat. No.ZM-0473); all purchased from ZSGB-BIO as ready-to-use working solutions, were applied and incubated at room temperature for 2 h. After three 3-min washes with phosphate-buffered saline (PBS), sections were incubated with enzyme-labeled goat anti-mouse/rabbit IgG polymer at 37 °C for 20 min. Antibody binding was then visualized with 3,3′-diaminobenzidine (DAB) for 5–8 min, followed by hematoxylin counterstaining for 20 s, differentiation of 1% hydrochloric acid (HCl) alcohol, and saturated lithium carbonate bluing. Finally, sections were dehydrated, cleared, and mounted for microscopic evaluation. For the evaluation of immunostaining, a combined scoring system based on staining intensity and the percentage of positive cells was used. Staining intensity was graded as 0 (no staining), 1 (weak), 2 (moderate), or 3 (strong). The percentage of positive cells was scored as 0 (<5%), 1 (5%–25%), 2 (26%–50%), 3 (51%–75%), or 4 (>75%). A final total score (range 0–12) was calculated by multiplying the intensity score by the percentage score.

### 2.6 Patient-derived organoid xenograft (PDOX) nude mouse model

All animal experiments were performed in strict compliance with the Guidelines for the Care and Use of Laboratory Animals of our institution, and the protocol was approved by the Institutional Animal Ethics Committee (Approval Number: TJFCH2023020). For each of the two biologically independent PDO lines (Case 1, Case 2, serving as biological replicates), we used 3 female 8 weeks-old nude mice (purchased from Tianjin International Joint Institute of Biomedicine) per PDO line, resulting in a total of 6 mice across the two lines. These mice served as technical replicates within each PDO line to account for intra-line variability. Organoids were dissociated into small clusters (50–200 μm) and enriched via centrifugation at 200 g for 5 min. The cell suspension was suspended in 100 µL of Matrigel mixed with equal volume of lung adenocarcinoma organoid medium (containing 1 × 10^7^ cells), and all procedures were performed on ice to prevent gelation. Nude mice were anesthetized with isoflurane, and injected subcutaneously into the interscapular area to form a unilateral gel droplet with 100 µL of the Matrigel-cell suspension. For each PDO line, a negative control group (n = 3 mice) receiving Matrigel blank injection (Matrigel: PBS = 1:1) without organoid cells was also included. Mice were individually housed and monitored daily for wound healing and general health. Negative control group (n = 3 mice) receiving Matrigel blank injection (Matrigel: PBS = 1:1) without organoid cells. Tumor dimensions were measured twice weekly with digital calipers, and volume was calculated as 0.5 × length × width^2^. Statistical comparisons between groups were performed using the mean tumor volume calculated from the three mice representing each PDO line, with the PDO line serving as the unit of biological replication.

### 2.7 Alamar blue™ cell viability assay

Organoids were retrieved using organoid recovery solution (Corning, 354248) to remove the Matrigel matrix, followed by dissociation with digestion solution and counting using a cell counter (Monwei, SmartCell). Cells were seeded into 96-well plates at a density of 3 × 10^3^ cells per well. Upon reaching 20–50 μm diameter, organoids were replaced with fresh lung adenocarcinoma organoid medium containing cisplatin (99.30%, HY-17394, MCE) or 20(S)-ginsenoside Rg3 (99.82%, HY-N0603, MCE) at serial concentrations (0.001, 0.01, 0.1, 1, 10 and 50 μmol/L), and combination groups at cisplatin: Rg3 ratios of 1:1, 2:1, or 4:1. For each drug treatment condition of the cells, three replicate wells were set up for testing. Stock solutions of 20(S)-ginsenoside Rg3 were prepared by dissolving in DMSO. Working solutions were generated by serial dilution in lung adenocarcinoma organoid medium immediately before each experiment, ensuring that the final DMSO concentration did not exceed 0.1% in any well. Cisplatin working solutions were freshly prepared in sterile water prior to each treatment. Vehicle-matched control groups were established for all experimental conditions. For Rg3, a DMSO-matched control (≤0.1%) was used; for cisplatin, drug-free culture medium served as control; and for combinations, a combined solvent control was included. After 48 h of drug treatment, an appropriate volume of AlamarBlue® reagent (Invitrogen, DAL1025) was added to each well. The optical density (OD, Ex/Em: 560/590 nm) of each well was measured using a microplate reader (Molecular Devices, Versamax) and the reduction rate was calculated. Dose-response curves were plotted, and the half-maximal inhibitory concentration (IC_50_) of each drug was calculated by nonlinear regression fitting. Meanwhile, the normalized Area Under the Dose-response Curve (AUC) was calculated.

### 2.8 Intracellular ROS detection

The organoid suspensions were collected and centrifuged at 200 g for 3 min, and resuspended in fresh medium containing 10 μmol/L DCFH-DA probe (Beyotime, S0033S). Samples were incubated at 37 °C in the dark for 30 min, washed three times, and fixed in 4% paraformaldehyde for 15 min in the dark. Nuclei were counterstained with antifade mounting medium with DAPI (Beyotime, P0131). Fluorescence images were acquired on a confocal microscope (Zeiss, LSM 880).

### 2.9 Terminal deoxynucleotidyl transferase dUTP nick end labeling (TUNEL) assay

Organoids treated with drugs were collected as described above and adjusted to a cell density of 1 × 10^6^ cells/mL. Samples were permeabilized with 0.5% Triton X-100 for 5 min and washed twice. The DAB-based TUNEL Apoptosis Detection Kit (Beyotime, C1091) was used according to the manufacturer’s instructions. Briefly, 50 μL of TUNEL reaction mixture was added to each sample, followed by incubation at 37 °C for 30 min. The reaction was terminated by washing with PBS, and DAB substrate was applied for 10 min. Nuclei were counterstained with hematoxylin, and samples were observed under a light microscope.

### 2.10 Statistical analysis

All data were obtained from three independent experiments with three replicates each, and results were presented as the mean ± standard deviation (S.D.). Statistical significance was analyzed using GraphPad Prism software (version 9.5.0) with one-way ANOVA or two-way ANOVA with Tukey’s multiple comparisons test as appropriate. Significance levels were indicated as follows: *p < 0.05, **p < 0.01, ***p < 0.001, ****p < 0.0001.

## 3 Results

### 3.1 Establishment and growth status evaluation of patient-derived lung adenocarcinoma organoid models

**FIGURE 1:**
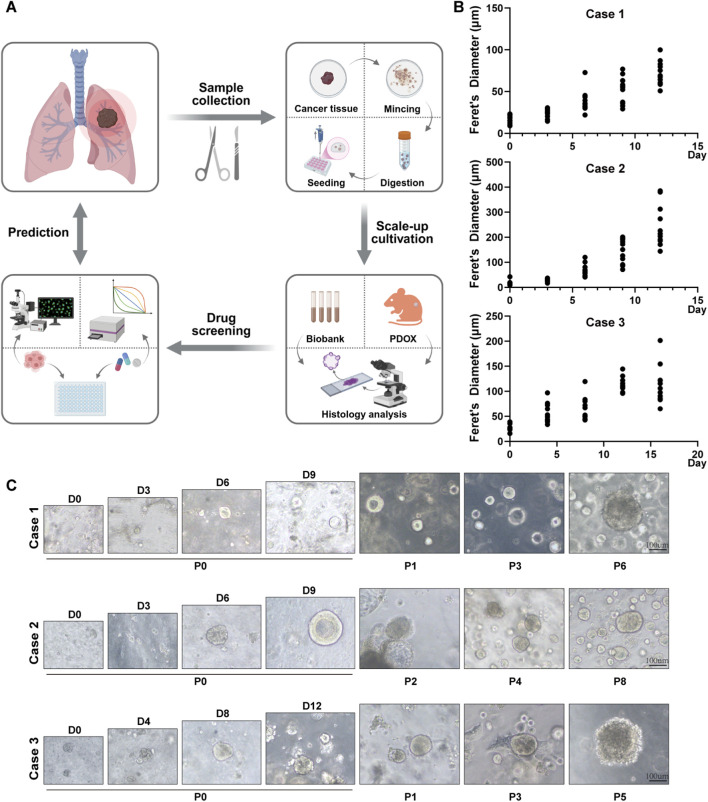
Establishment, growth analysis, and morphological characterization of lung adenocarcinoma organoids. **(A)** Schematic illustration of the lung adenocarcinoma organoid construction workflow. Surgical resected tumor tissues were mechanically dissociated and enzymatically digested, and resuspended in Matrigel for 3D culture. Successfully established lung adenocarcinoma organoids can be utilized for drug screening and mechanistic studies. **(B)** Statistical analysis of growth status of primary-cultured organoids derived from three lung adenocarcinoma cases. **(C)** Representative bright-field images in left panel illustrating morphological features of three primary-cultured lung adenocarcinoma organoid lines. The right panel displays morphological characteristics of organoids during long-term culture. Scale bar: 100 μm.

By optimizing culture conditions, we successfully established three patient-derived lung adenocarcinoma organoid lines from two surgical resection specimens and one malignant pleural effusion sample. The clinical characteristics of the corresponding patients are detailed in Table 1. As illustrated in Figure 1A, we established a standardized workflow for organoid construction, encompassing tumor tissue acquisition, processing, enzymatic digestion, cell culture and its application. Under these conditions, stable organoids visible under the light microscope generally emerged within 7–10 days.

**TABLE 1:** Clinical information of patients with three established lung adenocarcinoma organoids.

No.	Gender	Age	Tumor Site	Diagnosis	Tumor Size	Subtypes	TNM Stage	Metastasis
Case 1	Female	58	Right lower lobe	Invasive adenocarcinoma	4.0cm × 3.5cm × 3.0cm	Acinar 10% Papillary 60% Micropapillary 30%	T2aN2M0 Stage III	None
Case 2	Male	59	Right upper lobe	Invasive adenocarcinoma	4.0cm × 2.6cm × 1.9cm	Acinar Solid	T2aNxM1 Stage IV	Bone metastasis
Case 3	Female	37	Left upper lobe	Invasive adenocarcinoma	2.2cm × 1.3cm × 0.7cm	Acinar 45% Papillary 15% Lepidic 40%	T1cN0M0 Stage II	None

All three PDO lines exhibited consistent growth kinetics during cultivation. The number of organoids per field of view and their Feret’s diameters were quantified to generate growth curves (Figure 1B). Primary cultured organoids entered a rapid proliferation phase starting from day 8, reaching diameters of approximately 200 μm by day 12. Morphologically, primary organoids appeared as small cell clusters during the initial culture phase, and these spheroids predominantly exhibited solid or honeycomb-like architectures, with occasional hollow cystic formations by day 8, displaying clear boundaries and good refractivity. Each organoid line retained distinct structural characteristics reflecting its origin (Figure 1C). Following serial passaging, the organoids exhibited robust growth kinetics while preserving their characteristic 3D architecture, with no significant morphological drift observed.

### 3.2 Histomorphology validation of lung adenocarcinoma organoids

**FIGURE 2:**
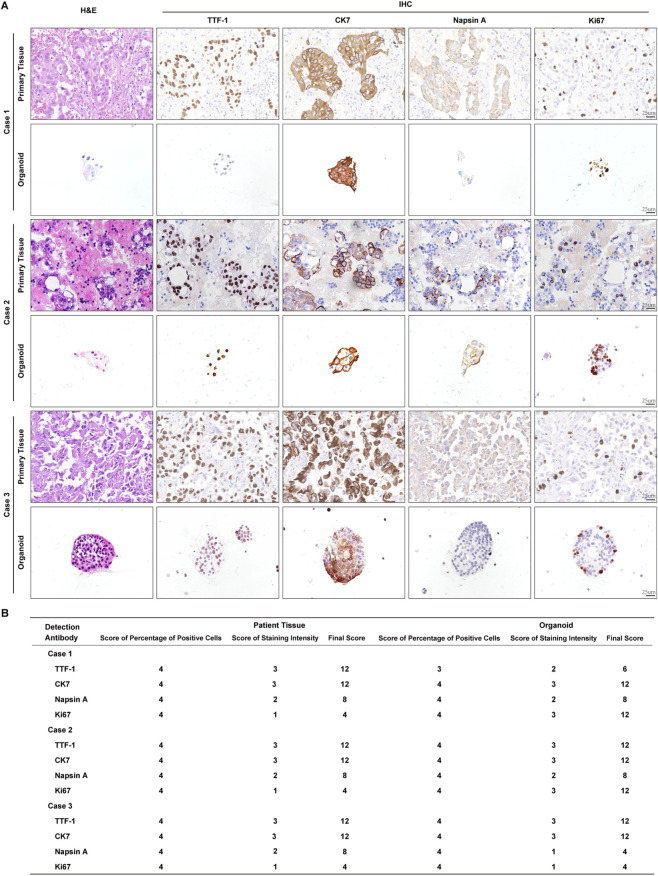
Morphological and immunophenotypic characterization of lung adenocarcinoma organoids. **(A)** Representative HE and IHC staining images comparing lung adenocarcinoma organoids with matched parental tumor tissues. Scale bar: 25 μm. **(B)** Triple-line chart showing quantitative scores of staining results.

Pathological analyzes were conducted to verify the histological fidelity of lung adenocarcinoma organoids. HE staining revealed that both organoids and parental tissues exhibited marked morphological and architectural heterogeneity, characterized by enlarged, hyperchromatic nuclei with an elevated nuclear-to-cytoplasmic ratio, arranged in glandular or solid architectural patterns (Figure 2A), indicating that the organoids faithfully recapitulated the pathological features of their corresponding primary lesions.

Immunohistochemical analysis demonstrated stable expression of lung adenocarcinoma-associated biomarkers in these organoids (Figure 2A). Ten random fields were evaluated for each marker. A combined score was calculated by multiplying the percentage of positive cells by the staining intensity (Figure 2B). Protein expression levels of TTF-1, CK7, Napsin A, and Ki67 in organoids were largely consistent with those of parental tumor tissues. Notably, the Ki67 positivity rate was significantly higher in lung adenocarcinoma organoids compared to their parental tissues (*p* < 0.01), suggesting enhanced proliferative capacity in these organoid models.

### 3.3 Establishment and characterization of the PDOX mouse model

**FIGURE 3:**
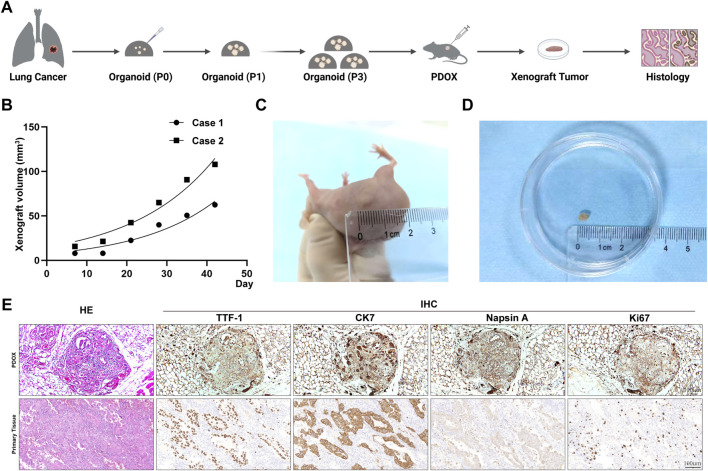
*In vivo* characterization of lung adenocarcinoma organoid-derived xenografts. **(A)** Schematic diagram of lung adenocarcinoma organoid-derived xenografts construction workflow. **(B)** Growth curves of lung adenocarcinoma organoid-derived xenografts. **(C)** The appearance of the xenografts as nodular masses protruding from the skin surface. **(D)** the xenografts dissected from the PDOX models exhibiting typical nodular morphology with clear boundaries. **(E)** Characterization of cellular morphology and expression of the lung adenocarcinoma-specific molecular markers (TTF-1, CK7, Napsin A, and Ki67) in organoid-derived xenografts and their parental tumor tissue. Scale bar: 100 μm.

To evaluate the *in vivo* tumorigenicity of the lung adenocarcinoma organoids and their ability to maintain biological characteristics, we established PDOX models in immunodeficient mice. As illustrated in Figure 3A, third-passage organoids were mixed with Matrigel and transplanted subcutaneously into the dorsal region of nude mice. The tumor growth curves revealed that xenografts entered a phase of rapid proliferation after the second week post-transplantation, reached a growth plateau at the sixth week, and were harvested for analysis at the seventh week (Figure 3B). The xenografts appeared as nodular masses protruding from the skin surface. Upon dissection, the tumors exhibited typical nodular morphology with clear boundaries with a mean volume of approximately 65 mm^3^ (Figures 3C,D).

Histopathological analysis confirmed that the xenografts highly recapitulated the architectural features of the original tumors. HE staining revealed that they maintained the characteristic acinar-like structures and cellular atypia of lung adenocarcinoma. Immunohistochemical profiling demonstrated that the xenografts expressed lung adenocarcinoma-specific biomarkers TTF-1, CK7, and Napsin A, with expression patterns and intensities closely matching those of the parental tumor tissues (Figure 3E). The successful establishment of this PDOX model validated the *in vivo* tumorigenic potential of these lung adenocarcinoma organoids.

### 3.4 Pharmacodynamic evaluation of Rg3 in enhancing chemosensitivity of lung adenocarcinoma organoids to cisplatin

**FIGURE 4:**
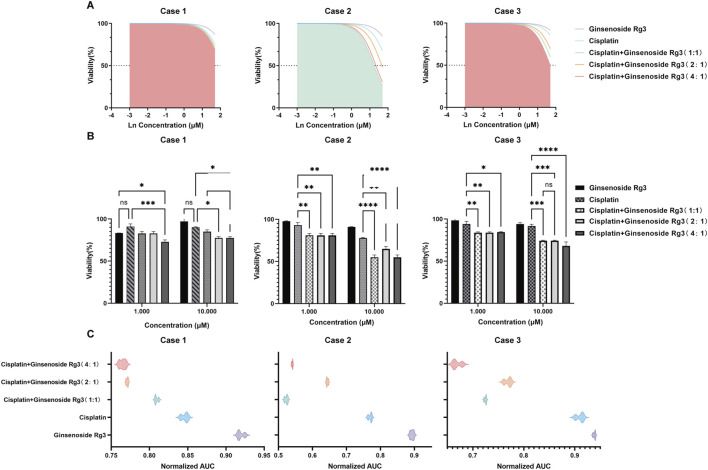
Rg3 enhancing chemotherapy sensitivity in lung adenocarcinoma organoids. **(A)** Organoid viability visualized using dose-response curves of different drug groups illustrating both IC_50_ determination and optimization of combination ratios. The color under the curve was filled with that of the group with the lowest cell activity. **(B)** Organoid viability under different treatment conditions visualized using bar graphs. **(C)** Normalized AUC analysis of pharmacodynamic efficacy. Data are presented as mean ± S.D. (n = 3). Statistical significance was determined by two-way ANOVA (factors: treatment and organoid line) with Tukey’s multiple comparisons test. Significance levels were indicated as follows: *p < 0.05, **p < 0.01, ***p < 0.001, ****p < 0.0001.

Next, we examined the effects of Rg3 and cisplatin, both individually and in combination, on these organoids. Morphologically, the organoids exhibited structural disruption, manifested as disintegration of the spherical architecture, loss of well-defined borders, and the presence of abundant cellular debris. Based on a wide-range screening across six concentrations (0.001–50 μM), 1 μM and 10 μM of the drugs were selected for subsequent combination experiments. Nonlinear regression analysis was employed to determine IC_50_ values across treatment groups, and the filled area under the curve represents the group with the strongest inhibitory effect (Figure 4A). The 4:1 ratio combination demonstrated the most potent inhibition (lowest IC_50_) in samples 1 and 3, whereas the strongest inhibitory effect in sample 2 was observed at the 1:1 ratio, indicating that organoids derived from different sources exhibited differential responses to the same drug combination.

Furthermore, a bar graph was used to visually present the effects of different drug combinations on organoid viability (Figure 4B). As shown, the organoid survival rate in the combination treatment group was lower than that in the monotherapy groups. This difference was more pronounced at a drug concentration of 10 μM compared to 1 μM, suggesting that Rg3 may have the potential to enhance the chemotherapeutic efficacy of cisplatin. The normalized area under the dose-response curve (AUC) was calculated to provide an assessment of the pharmacodynamic efficacy (Figure 4C). Consistent with the drug sensitivity trends revealed by the IC_50_ values, the AUC results similarly reflected the relative potency of the drugs, and together they provided complementary assessments of drug efficacy.

### 3.5 Effects of Rg3 and cisplatin combination on apoptosis and ROS levels in lung adenocarcinoma organoids

**FIGURE 5:**
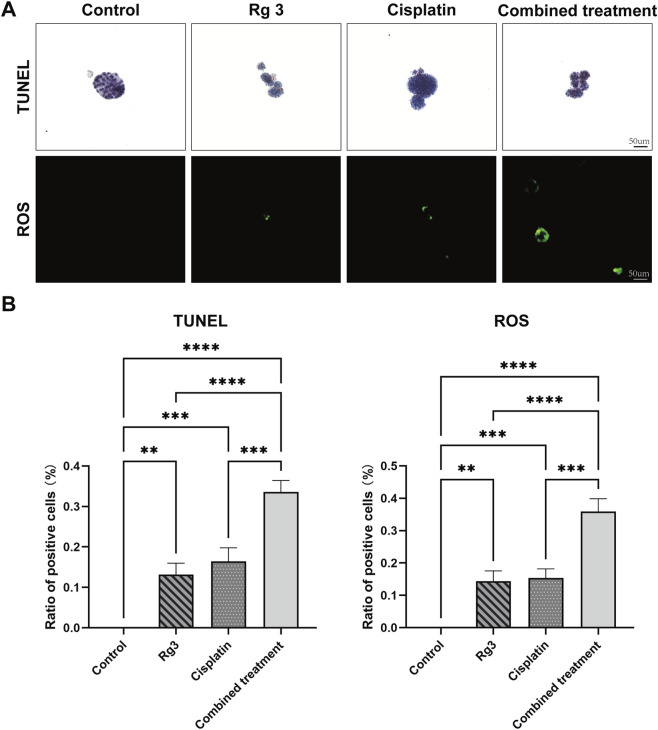
Rg3 enhancing chemosensitivity of lung adenocarcinoma organoids to cisplatin via the apoptosis pathway. **(A)** TUNEL staining and ROS assay among the drug-treated groups illustrating a significant increased proportion of apoptotic cells and ROS levels in the combination of cisplatin and Rg3 compared to the single drug group. **(B)** Statistical results of the proportion of apoptotic cells using TUNEL test were illustrated in left panel, and ROS levels were shown in the right panel. Scale bar: 50 μm. Data are presented as mean ± S.D. (n = 3). Statistical significance was determined by one-way ANOVA with Tukey’s multiple comparisons test. Significance levels were indicated as follows: *p < 0.05, **p < 0.01, ***p < 0.001, ****p < 0.0001.

To elucidate the specific mechanisms underlying the aforementioned chemosensitivity results, we conducted further investigations on different drug-exposed groups. We assessed apoptosis using the TUNEL assay. Quantitative data revealed a notable increase in the number of brown-stained apoptotic cells in the cisplatin plus Rg3 combination group when compared with the single-agent groups, indicating its pro-apoptotic effect (Figures 5A,B).

Furthermore, we explored the role of ROS in this process. Compared with the control group, the fluorescence intensity was significantly higher in the cisplatin plus Rg3 group (Figures 5A,B), suggesting an elevation in intracellular ROS levels, which may be associated with the activation of apoptotic pathways.

These results collectively demonstrate a correlation between the combination treatment and increased ROS levels and apoptosis, suggesting a potential role for ROS in the observed effects, though the underlying mechanisms require further validation.

## 4 Discussion

In this study, we successfully established three patient-derived lung adenocarcinoma organoid models and the *in vivo* organoid xenograft models that faithfully recapitulated the histological architecture and molecular phenotype of the corresponding parental tumors, aligning with the findings of Kim *et al.*, who have reported that lung-cancer organoids can preserve the genomic landscape of the original lesions (Kim et al., 2019). The Ki67 index is directly associated with cellular proliferative activity. In this study, it was observed that the Ki67 expression level in the organoids was significantly higher than that in their parental tissues of origin. This suggests that the *in vitro* culture conditions may have selected for or promoted the expansion of cell populations with high proliferative potential. Furthermore, our organoid models maintained stable three-dimensional structural integrity and proliferative capacity during continuous passaging, and its long-term culture stability (Wang et al., 2023).To further support the biological relevance of our models, we successfully established corresponding PDX models, confirming histological consistency across PDOs, PDX, and primary tumors. All organoids used underwent pathological review, demonstrating conserved morphology and protein expression profiles relative to the original tissues. While we acknowledge that systematic multi-omics comparison remains the gold standard for evaluating model fidelity—and we have not yet completed comprehensive genomic/transcriptomic sequencing of paired samples due to project constraints—these rigorous phenotypic and functional validations provide a solid foundation for the subsequent pharmacodynamic investigations conducted in this study.

We employed these organoid models to evaluate the antitumor effects of ginsenoside Rg3, both as a monotherapy and in combination with cisplatin. Pharmacodynamic evaluation demonstrated that the combination treatment exerted superior inhibitory effects compared to either agent alone. Notably, organoids derived from different patients exhibited varied optimal combination ratios for achieving the strongest response, highlighting the underlying tumor heterogeneity and underscoring the unique value of organoid models in personalized drug sensitivity testing (Tang and Tian, 2026; Taverna et al., 2024). We note that the observed enhancement of inhibitory activity and reduction in the IC_50_ of cisplatin in combination treatment represent preliminary pharmacological trends. To definitively characterize the nature of this drug interaction (e.g., synergistic, additive, or antagonistic), future studies employing systematic dose-matrix designs and quantitative analyses—such as Combination Index (Chou-Talalay) or Bliss independence model calculations—are required. Furthermore, regarding the cisplatin concentrations used in our *in vitro* assays, we selected a broad range (0.001–50 µM) primarily to ensure robust dose-response characterization. It is important to clarify that the key findings of enhanced efficacy in combination with Rg3 were consistently observed at clinically relevant concentrations (1 μM and 10 µM), which align with reported peak plasma levels in patients. The higher concentrations (e.g., 50 µM) were included for technical completeness of the sigmoidal curve fitting and do not form the basis of our core conclusions. We acknowledge that *in vitro* sensitivity in organoid models, which lack systemic pharmacokinetics, must be interpreted with appropriate translational bridging to clinical pharmacodynamics (PK/PD). We also recognize the absence of normal lung organoid or epithelial controls as a limitation in assessing therapeutic selectivity. While published studies suggest that cisplatin at our tested concentrations (1–10 µM) exhibits limited toxicity to normal lung organoids (Urien and Lokiec, 2004), and that Rg3 may possess antioxidant and protective effects on lung tissue at low micromolar concentrations (Li et al., 2017; Qiu et al., 2019), these observations are indirect. Future studies incorporating normal human bronchial epithelial organoids or primary epithelial cells are essential to systematically evaluate the toxicity-selectivity profile of this combination regimen. Collectively, these findings suggest that Rg3 may provide experimental support for personalizing treatment strategies in patients with platinum-insensitive or platinum-resistant lung adenocarcinoma.

In the present study, treatment with the combination of Rg3 and cisplatin was observed to induce an increased proportion of apoptotic cells and elevated levels of ROS in organoids. These changes may be associated with its potential chemosensitizing effect. However, chemoresistance in lung adenocarcinoma represents a networked issue involving multiple intertwined pathways, encompassing complex mechanisms such as abnormal activation of various signaling cascades, the epithelial-mesenchymal transition (EMT) process, and cellular metabolic reprogramming (Zhou et al., 2019; Fan et al., 2024; Du et al., 2025; Sun et al., 2025). Therefore, the sensitizing effect of Rg3 is likely not attributable to the inhibition of a single target but rather reflects the characteristic “multi-target, system-level regulatory” capacity unique to active components derived from traditional Chinese medicine (Hao et al., 2017; Hao et al., 2015).

The ROS burst triggered by Rg3 may serve as a crucial network-perturbing signal contributing to this effect. On the one hand, ROS can directly impair mitochondrial function and initiate the intrinsic apoptotic pathway (Hwang et al., 2022; Zhang et al., 2025), which aligns with the observed increase in TUNEL-positive cells in our study. On the other hand, as an important secondary messenger, ROS may simultaneously radiate to regulate multiple parallel pathways associated with drug resistance (Nazar et al., 2024). For example, excessive ROS can inhibit the activity of the PI3K/AKT survival signaling axis, thereby weakening the anti-apoptotic capacity of tumor cells (Wei et al., 2022; Kuttikrishnan et al., 2025; Teng et al., 2025). Concurrently, it may also reverse or suppress the EMT process, which is closely linked to tumor cell invasion, stemness, and drug resistance. Previous studies have suggested that Rg3 can block EMT by inhibiting NF-κB, thereby sensitizing cells to chemotherapy (Wang et al., 2018). This coordinated modulation of the ROS-apoptosis axis, survival signaling, and cellular phenotype outlines a picture in which Rg3 rebalances the signaling network of tumor cells in a systems biology manner, consistent with the modern pharmacological understanding of the multi-component, multi-target action mode of traditional Chinese medicine (Du et al., 2026). We acknowledge that our mechanistic understanding remains preliminary compared to the depth of signaling analyses in fields like cardiovascular research (Liu et al., 2025). Future work must systematically identify the specific apoptotic regulators (e.g., cleaved caspases, Bcl-2 dynamics) involved and delineate Rg3’s crosstalk with core resistance pathways. To precisely test the hypothesis that Rg3 modulates mitochondrial electron transport, employing advanced tools—such as biosensors for detecting redox modifications in respiratory complexes (Hao et al., 2026), will be crucial to determine if chemosensitization occurs via altered redox states and post-translational modifications of the electron transport chain. We emphasize that the association between ROS elevation and enhanced apoptosis in our model is currently correlative. To establish a causal link, functional validation—such as rescue experiments with ROS scavengers and direct measurements of mitochondrial membrane potential and cytochrome c release—will be essential in future studies. Future research must move beyond correlative observations and focus on mapping its specific signaling network. For instance, it would be valuable to investigate whether Rg3 precisely modulates electron transport chain function and cellular redox homeostasis by regulating oxidative modifications of mitochondrial Complex I, and to elucidate its crosstalk with core apoptotic proteins and known resistance pathways (Hao et al., 2026). The currently observed synergistic trend remains preliminary, and such in-depth mechanistic analysis, coupled with rigorous drug interaction assessment, will establish a solid theoretical foundation for developing Rg3 as a novel chemosensitizing agent.

We fully acknowledge the methodological and interpretative limitations of this study. Firstly, the conclusions are drawn from a focused set of three lung adenocarcinoma PDO lines. While these models were rigorously validated phenotypically and functionally, the limited sample size and the homogeneity in subtype (all adenocarcinomas) constrain the broad generalizability of the findings. Secondly, the absence of paired multi-omics data (genomic/transcriptomic) for the PDOs and their parental tumors represents a key technical limitation, preventing a definitive assessment of model fidelity at the molecular level and a deeper exploration of genotype-phenotype correlations in the drug response. Thirdly, our PDOs were cultured in a Matrigel-based three-dimensional system. While standard and effective for PDO establishment, this murine-derived matrix lacks the tailored biochemical and biophysical cues of a fully defined, biomimetic scaffold, which may limit the recapitulation of critical tumor microenvironment (TME) complexity. Recent advances in tissue engineering, such as the development of injectable extracellular matrix (ECM)-mimicking microtissues for regenerative medicine (Yao et al., 2026), highlight the potential of customized biomaterials to better preserve native tissue architecture and signaling networks. Future integration of similar biomimetic scaffolds into PDO culture could enhance model fidelity and provide a more physiologically relevant context for investigating mechanisms of drug response, including Rg3-enhanced inhibitory activity. Additionally, our Matrigel-based PDOs model, although superior to conventional two-dimensional cultures, lacks critical components of the native tumor immune microenvironment, such as immune cells and cytokine networks (Ou et al., 2023). This precludes the investigation of potential immunomodulatory effects of Rg3 and limits the physiological completeness of the model. Incorporating tailored biomimetic scaffolds or co-culture systems represents a crucial future direction for enhancing microenvironmental fidelity (Wang et al., 2025; Sun et al., 2023). Furthermore, the observed reduction in the half-maximal inhibitory concentration (IC_50_) in the combination treatment group represents only a preliminary trend toward enhanced efficacy, based on a fixed-ratio design. The precise nature of the drug interaction—whether synergistic, additive, or antagonistic—requires future validation through systematic and quantitative analyzes using established models (e.g., Combination Index or Bliss independence). Finally, while we identified a correlation between Rg3 treatment and increased levels of ROS and apoptosis, our mechanistic exploration remains at a phenomenological level. Direct functional evidence, including rescue experiments with ROS scavengers and assessment of key mitochondrial apoptotic events, is needed to substantiate causality. Compared with the detailed signaling pathway dissection achieved in other fields (Zhong et al., 2025), our understanding of the specific upstream and downstream regulators and their crosstalk with established resistance pathways, such as PI3K/AKT signaling and EMT remain incomplete. These limitations clearly delineate essential experimental pathways for future validation and more in-depth mechanistic elucidation.

Despite these limitations, this platform provides experimental support for the preliminary *in vitro* screening of integrated traditional Chinese and Western medicine regimens. We emphasize that the observed enhancement of cisplatin activity by Rg3 represents preliminary pharmacological trends, and the precise nature of this drug interaction—whether synergistic, additive, or antagonistic—remains to be formally determined. We also acknowledge that, due to limitations in project funding and the current study timeline, we were unable to perform comprehensive molecular profiling (e.g., major driver mutations, DNA repair pathway markers, or baseline ROS/antioxidant capacity) that would help correlate PDO characteristics with cisplatin response and explain the differential optimal combination ratios observed across different cases. This represents an important direction for future investigation. In future studies, we aim to further clarify the relationship between drug screening outcomes in organoids and clinical pharmacologic parameters, thereby facilitating its application in translational medicine. A critical and immediate step will be the implementation of matched multi-omics validation among patients-PDO-primary tumors. Additionally, to deepen the mechanistic understanding, future work will prioritize functional validation of the ROS-mediated pathway, including rescue experiments and direct measurement of mitochondrial dysfunction, to move from correlation to causation, and will involve adopting more granular analytical approaches to dissect the specific molecular network through which Rg3 rebalances tumor cell signaling. Systematic dose-matrix designs and quantitative interaction analyses (e.g., Combination Index or Bliss independence models) will be essential to definitively characterize the interaction profile. The scope of investigation must be expanded to encompass a broader range of lung cancer subtypes. Through these refinements, the platform holds promise for delivering more precise and feasible individualized treatment options for lung cancer patients who are unresponsive to or develop resistance against standard therapeutic regimens.

## Data Availability

The datasets presented in this study can be found in online repositories. The names of the repository/repositories and accession number(s) can be found in the article/supplementary material.
